# Supplementary material for: Socio-economic gradients in hypertension and diabetes management amid the COVID-19 pandemic in India
Source: PLoS One. 2025 Apr 2;20(4):e0315867. doi: 10.1371/journal.pone.0315867 (PMC11964223; doi:10.1371/journal.pone.0315867)
Supplement: S1 Appendix — (PDF) [file pone.0315867.s001.pdf]

Supplementary file

A Results before the pandemic

The following Tables A.1 and A.2 show the results before the pandemic.

Table A.1: Dissimilarity index for hypertension across age groups before the pandemic

**Hypertension**	**Hypertension prevalence**			**Hypertension medication**		
	Estimates	95 percent CIs	Proportion	Estimates	95 percent CIs	Proportion
**Age below 30**						
Dissimilarity index	0.17***	(0.155,0.185)	1.000	0.267***	(0.222,0.311)	1.000
Gender	0.127***	(0.111,0.143)	0.747	0.185***	(0.137,0.234)	0.696
Caste and tribe	0.009***	(0.003,0.014)	0.050	0.011*	(0,0.022)	0.041
Wealth	0.012***	(0.006,0.019)	0.072	0.013*	(0,0.025)	0.047
Education	0.005*	(-0.001,0.011)	0.030	0.01	(-0.002,0.023)	0.039
Location of living	0.017***	(0.012,0.023)	0.101	0.047***	(0.024,0.071)	0.177
**Age 30-39**						
Dissimilarity index	0.119***	(0.108,0.13)	1.000	0.231***	(0.197,0.266)	1.000
Gender	0.057***	(0.044,0.069)	0.475	0.167***	(0.131,0.204)	0.723
Caste and tribe	0.013***	(0.009,0.018)	0.113	0.013**	(0.001,0.026)	0.058
Wealth	0.021***	(0.015,0.027)	0.176	0.01*	(-0.003,0.023)	0.043
Education	0.011***	(0.008,0.015)	0.096	0.016**	(0.003,0.03)	0.071
Location of living	0.017***	(0.012,0.022)	0.140	0.024***	(0.009,0.04)	0.105
**Age 40-49**						
Dissimilarity index	0.07***	(0.062,0.079)	1.000	0.179***	(0.152,0.205)	1.000
Gender	0.019***	(0.013,0.026)	0.276	0.086***	(0.061,0.112)	0.483
Caste and tribe	0.007***	(0.002,0.012)	0.101	0.019***	(0.006,0.033)	0.109
Wealth	0.022***	(0.016,0.028)	0.310	0.035***	(0.02,0.049)	0.196
Education	0.01***	(0.006,0.014)	0.144	0.006**	(0,0.011)	0.032
Location of living	0.012***	(0.007,0.016)	0.170	0.032***	(0.018,0.046)	0.181
**Age 50-59**						
Dissimilarity index	0.064***	(0.055,0.073)	1.000	0.188***	(0.168,0.208)	1.000
Gender	0.001	(-0.001,0.003)	0.023	0.057***	(0.041,0.072)	0.301
Caste and tribe	0.008***	(0.004,0.012)	0.132	0.027***	(0.017,0.038)	0.145
Wealth	0.029***	(0.023,0.036)	0.456	0.056***	(0.043,0.069)	0.299
Education	0.009***	(0.005,0.013)	0.141	0.011***	(0.005,0.016)	0.057
Location of living	0.016***	(0.011,0.021)	0.249	0.037***	(0.027,0.048)	0.199
**Age 60+**						
Dissimilarity index	0.055***	(0.049,0.061)	1.000	0.172***	(0.16,0.185)	1.000
Gender	0.006***	(0.003,0.009)	0.104	0.023***	(0.016,0.03)	0.131
Caste and tribe	0.013***	(0.009,0.017)	0.240	0.023***	(0.017,0.03)	0.136
Wealth	0.019***	(0.016,0.023)	0.354	0.055***	(0.046,0.063)	0.317
Education	0.007***	(0.004,0.009)	0.126	0.022***	(0.016,0.029)	0.129
Location of living	0.01***	(0.007,0.013)	0.175	0.049***	(0.04,0.059)	0.287

Note: 95 percent confidence intervals (CIs) are calculated by bootstrap with 200 repetitions. Contributory categories are defined as follows: 1) Gender: male (binary variable), 2) Caste and Tribe: scheduled caste, scheduled tribe, and other backward class, 3) Wealth: wealth quintiles, 4) Education: primary, secondary, and higher educational achievement, 5) Location of living: urban and regional (binary variables). Pre-pandemic period: June 17, 2019 - January 30, 2020. Pandemic period: October 12, 2020 - May 20, 2021. $^{*}p < 0.1,^{**} p < 0.05,^{***} p < 0.01$

Table A.2: Dissimilarity index for diabetes across age groups before the pandemic

**Diabetes**	**Diabetes prevalence**			**Diabetes medication**		
	Estimates	95 percent CIs	Proportion	Estimates	95 percent CIs	Proportion
**Age below 30**						
Dissimilarity index	0.183***	(0.13,0.236)	1.000	0.087***	(0.045,0.128)	1.000
Gender	0.002	(-0.012,0.016)	0.011	0.016	(-0.004,0.036)	0.189
Caste and tribe	0.041**	(0.005,0.078)	0.225	0.009	(-0.01,0.027)	0.100
Wealth	0.052***	(0.012,0.093)	0.286	0.021*	(0,0.043)	0.245
Education	0.009	(-0.012,0.029)	0.047	0.003	(-0.014,0.021)	0.039
Location of living	0.079***	(0.027,0.131)	0.431	0.037**	(0.006,0.068)	0.426
**Age 30-39**						
Dissimilarity index	0.207***	(0.174,0.24)	1.000	0.072***	(0.045,0.1)	1.000
Gender	0.004	(-0.004,0.013)	0.021	0.021*	(0,0.042)	0.292
Caste and tribe	0.021*	(0,0.042)	0.100	0.005	(-0.007,0.018)	0.073
Wealth	0.036***	(0.015,0.057)	0.173	0.01	(-0.005,0.026)	0.140
Education	0.035***	(0.013,0.057)	0.170	0.005	(-0.006,0.015)	0.064
Location of living	0.111***	(0.081,0.14)	0.535	0.031***	(0.007,0.055)	0.431
**Age 40-49**						
Dissimilarity index	0.245***	(0.217,0.273)	1.000	0.059***	(0.038,0.079)	1.000
Gender	0.015***	(0.004,0.027)	0.063	0.002	(-0.007,0.011)	0.037
Caste and tribe	0.015***	(0.006,0.024)	0.061	0.022***	(0.006,0.037)	0.371
Wealth	0.076***	(0.059,0.094)	0.311	0.01	(-0.003,0.024)	0.175
Education	0.051***	(0.036,0.066)	0.209	0.009	(-0.003,0.021)	0.160
Location of living	0.087***	(0.066,0.109)	0.356	0.015*	(-0.001,0.031)	0.256
**Age 50-59**						
Dissimilarity index	0.285***	(0.26,0.31)	1.000	0.059***	(0.042,0.076)	1.000
Gender	0.006**	(0,0.011)	0.020	0.001	(-0.003,0.005)	0.020
Caste and tribe	0.026***	(0.017,0.034)	0.090	0.006	(-0.003,0.014)	0.095
Wealth	0.089***	(0.071,0.106)	0.310	0.011**	(0.002,0.02)	0.189
Education	0.057***	(0.044,0.07)	0.201	0.016***	(0.005,0.026)	0.262
Location of living	0.108***	(0.091,0.125)	0.378	0.026***	(0.014,0.038)	0.433
**Age 60+**						
Dissimilarity index	0.262***	(0.24,0.285)	1.000	0.053***	(0.04,0.067)	1.000
Gender	0.002	(-0.002,0.005)	0.006	0.004	(-0.002,0.009)	0.072
Caste and tribe	0.028***	(0.02,0.036)	0.108	0.006**	(0.001,0.012)	0.114
Wealth	0.075***	(0.062,0.089)	0.288	0.012***	(0.005,0.019)	0.231
Education	0.062***	(0.049,0.074)	0.236	0.01***	(0.003,0.017)	0.193
Location of living	0.095***	(0.08,0.11)	0.362	0.021***	(0.013,0.029)	0.390

Note: 95 percent confidence intervals (CIs) are calculated by bootstrap with 200 repetitions. Contributory categories are defined as follows: 1) Gender: male (binary variable), 2) Caste and Tribe: scheduled caste, scheduled tribe, and other backward class, 3) Wealth: wealth quintiles, 4) Education: primary, secondary, and higher educational achievement, 5) Location of living: urban and regional (binary variables). Pre-pandemic period: June 17, 2019 - January 30, 2020. Pandemic period: October 12, 2020 - May 20, 2021. $^{*}p < 0.1, ^{**}p < 0.05, ^{***}p < 0.01$

B Graphical representation of the D-Index

Figures B.1 and B.2 display the sorted predicted probabilities for hypertension and its treatment before and during the pandemic across age groups. The horizontal axis represents the fractional rank of probability size, accounting for differences in sample sizes across health outcomes. Since variations in predicted probabilities are driven solely by differences in covariates, the disparities between individual predicted probabilities reflect inequality associated with these factors. The shaded area denotes the difference between each predicted probability and the mean probability, marked by the red horizontal line. The total shaded area measures the absolute disparity of individual predicted probabilities from the mean, from which the D-index is calculated. Figures B.3 and B.4 show the sorted predicted probabilities for diabetes and its medication across the age groups.

Figure B.1: Predicted probabilities for hypertension prevalence

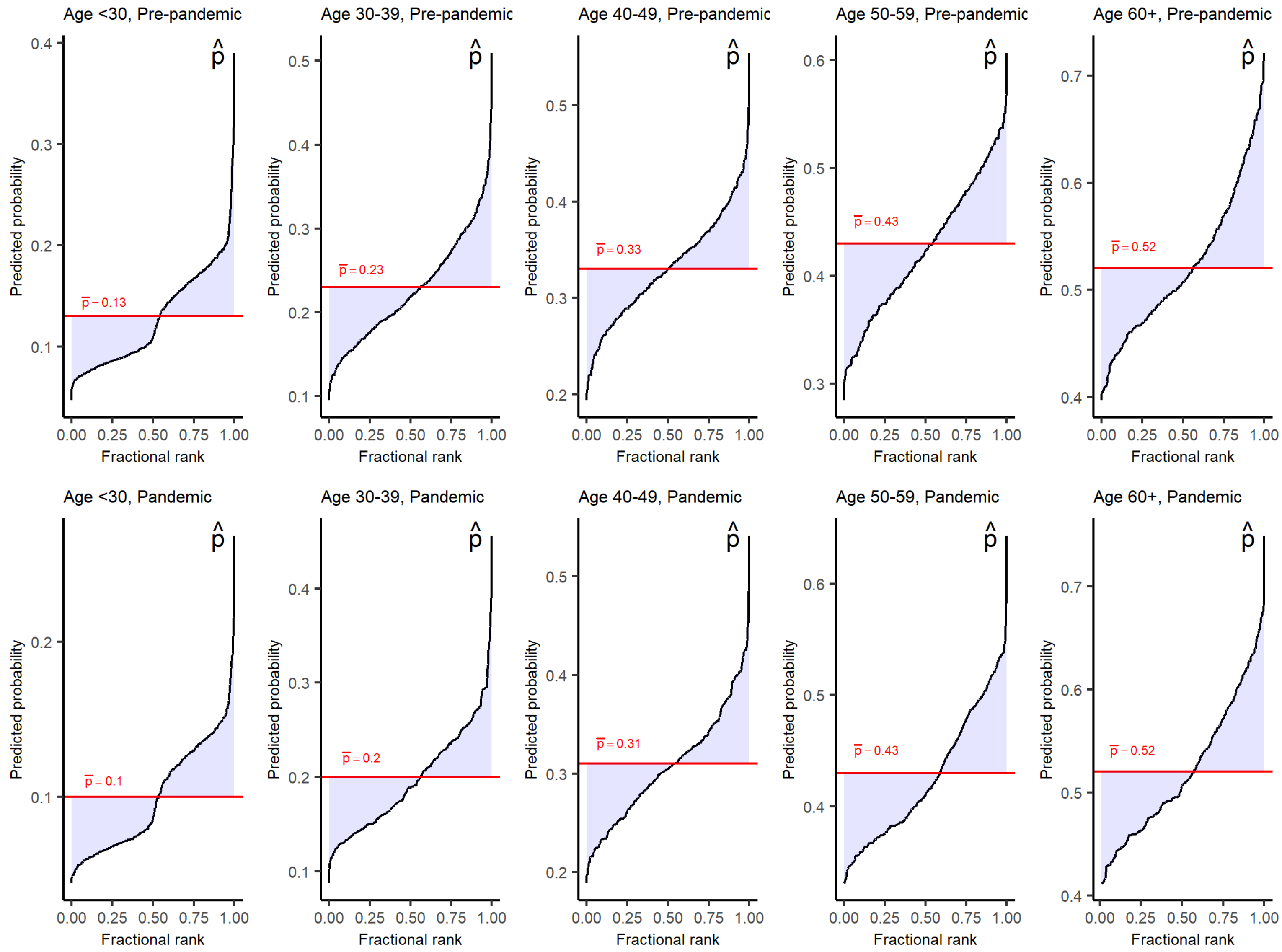

Note: The sorted predicted probabilities are plotted. The shaded areas indicate the difference between the predicted probability and the mean probability. Pre-pandemic period: June 17, 2019 - January 30, 2020. Pandemic period: October 12, 2020 - May 20, 2021.

Figure B.2: Predicted probabilities for hypertension treatment

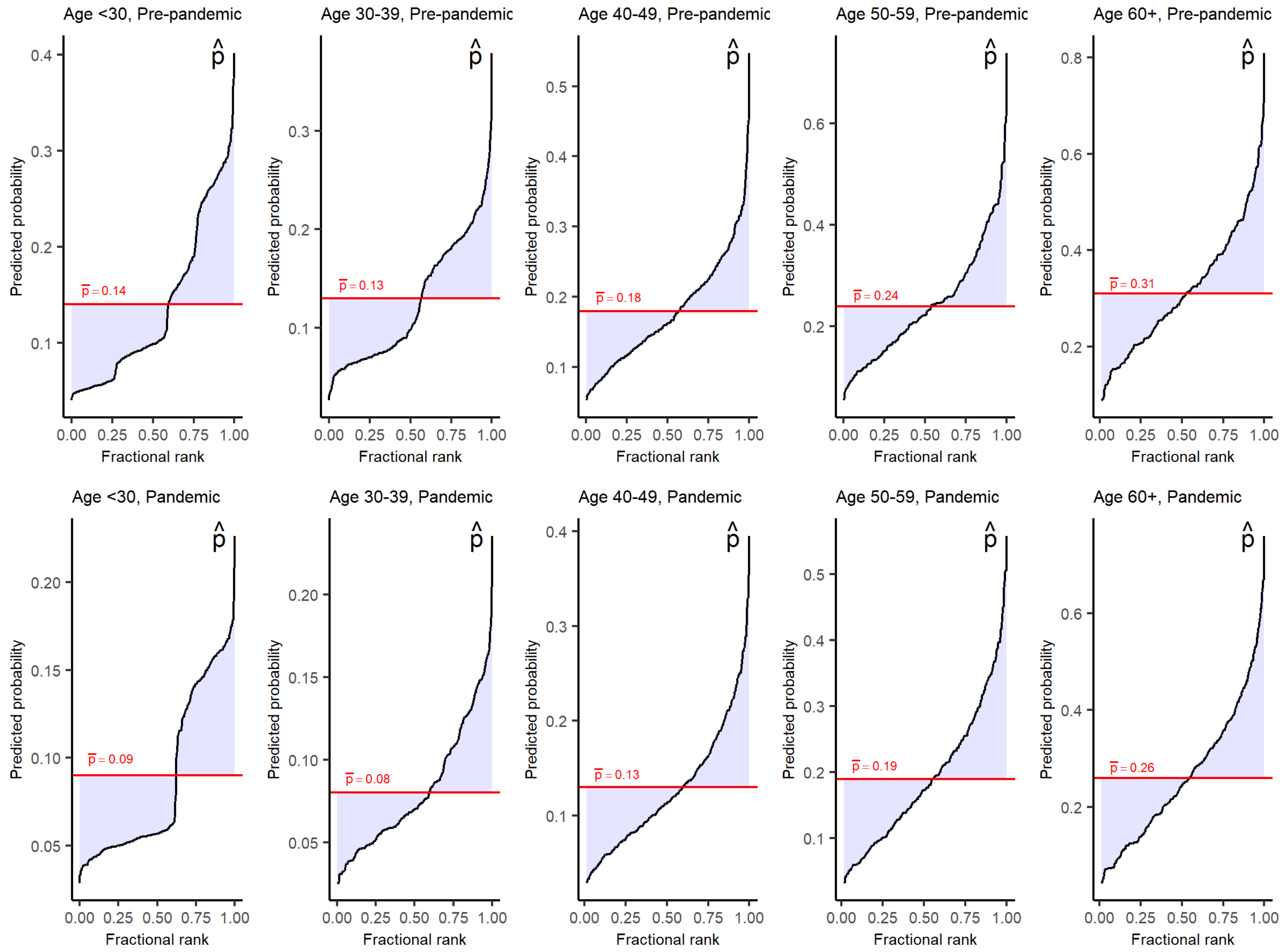

Note: The sorted predicted probabilities are plotted. The shaded areas indicate the difference between the predicted probability and the mean probability. Pre-pandemic period: June 17, 2019 - January 30, 2020. Pandemic period: October 12, 2020 - May 20, 2021.

Figure B.3: Predicted probabilities for diabetes prevalence

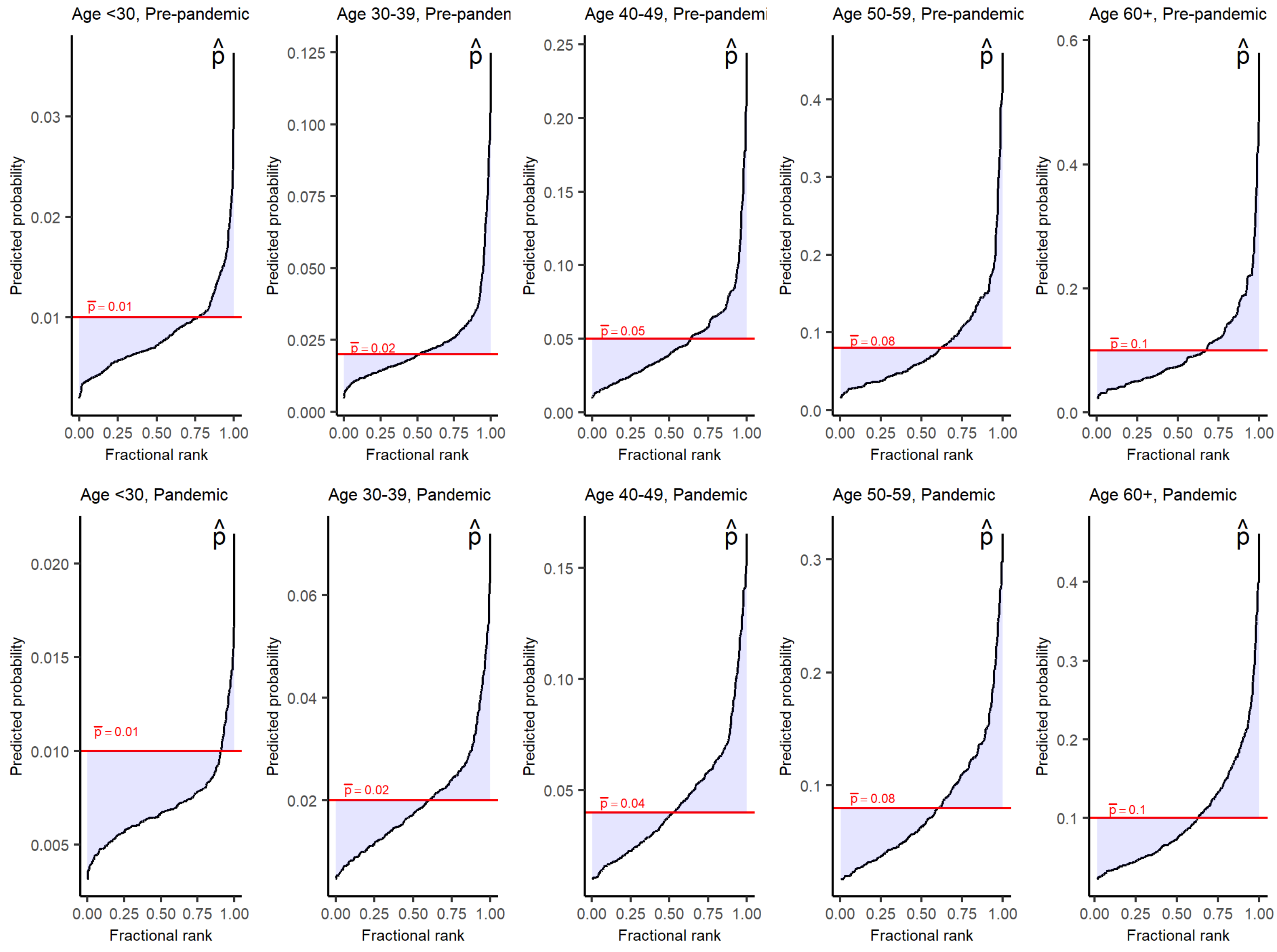

Note: The sorted predicted probabilities are plotted. The shaded areas indicate the difference between the predicted probability and the mean probability. Pre-pandemic period: June 17, 2019 - January 30, 2020. Pandemic period: October 12, 2020 - May 20, 2021.

Figure B.4: Predicted probabilities for diabetes treatment

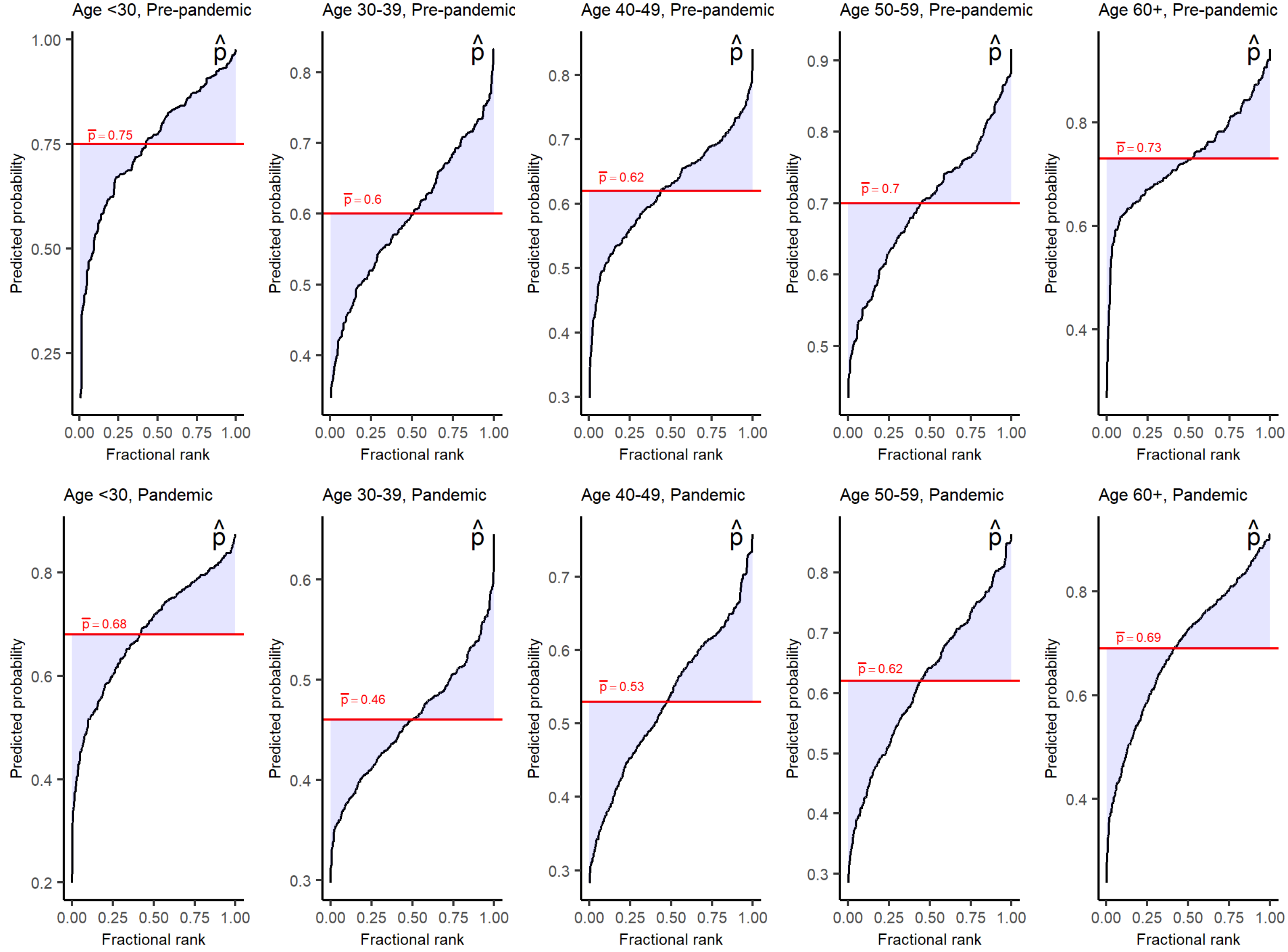

Note: The sorted predicted probabilities are plotted. The shaded areas indicate the difference between the predicted probability and the mean probability. Pre-pandemic period: June 17, 2019 - January 30, 2020. Pandemic period: October 12, 2020 - May 20, 2021.

C Results before the pandemic with the NFHS-4

This section provides additional results based on the survey conducted in 2015–2016 (NFHS-4) for reference purposes. In NFHS-4, individuals aged 15 to 49 were asked about their use of medication for hypertension. Therefore, the results include data on hypertension prevalence and medication only for those aged below 30, between 30 and 39, and between 40 and 49. The following 11 States are included in the analysis: Tamil Nadu, Arunachal Pradesh, Jharkhand, Odisha, Chhattisgarh, Madhya Pradesh, Uttarakhand, Haryana, Punjab, Rajasthan and Uttar Pradesh.

Table C.1: Regression analysis for hypertension prevalence and its treatment

	(1) Hypertension prevalence pre-pandemic period		(2) Hypertension medication pre-pandemic period	
Odds ratio				
Male	1.479***	[1.434,1.527]	0.415***	[0.381,0.452]
Age 30-39	2.243***	[2.173,2.314]	0.816***	[0.757,0.880]
Age 40-49	4.109***	[3.981,4.241]	1.000	[0.929,1.076]
Scheduled caste	0.882***	[0.849,0.915]	0.995	[0.915,1.081]
Scheduled tribe	1.034*	[0.994,1.076]	0.684***	[0.622,0.753]
Other backward class	0.903***	[0.876,0.930]	0.960	[0.899,1.024]
Wealth 2nd quintile	1.016	[0.978,1.055]	1.297***	[1.175,1.431]
Wealth 3rd quintile	1.144***	[1.100,1.190]	1.559***	[1.412,1.722]
Wealth 4th quintile	1.306***	[1.251,1.363]	1.770***	[1.594,1.965]
Wealth 5th quintile	1.403***	[1.336,1.473]	2.058***	[1.834,2.309]
Primary education	0.945***	[0.919,0.973]	1.150***	[1.078,1.227]
Secondary education	0.846***	[0.805,0.888]	1.023	[0.914,1.146]
Higher education	0.782***	[0.747,0.819]	1.181***	[1.066,1.308]
Urban	1.058***	[1.029,1.089]	1.004	[0.943,1.070]
Southern	1.150***	[1.106,1.197]	1.193***	[1.090,1.307]
Northern	1.302***	[1.255,1.351]	1.588***	[1.469,1.716]
North eastern	1.503***	[1.438,1.572]	0.972	[0.874,1.080]
Eastern	1.056***	[1.024,1.089]	1.542***	[1.436,1.656]
State fixed effect	Yes		Yes	
Observations	251903		36690	

Exponentiated coefficients; 95% confidence intervals in brackets

Standard errors are robust to heteroskedasticity

Pre-pandemic period: January 20, 2015 - December 04, 2016

* $p < 0.1$, ** $p < 0.05$, *** $p < 0.01$

Table C.2: Dissimilarity index for hypertension across age groups before the pandemic

Hypertension	Hypertension prevalence			Hypertension medication		
	Estimates	95 percent CIs	Proportion	Estimates	95 percent CIs	Proportion
**Age below 30**						
Dissimilarity index	0.119***	(0.109,0.129)	1.000	0.143***	(0.124,0.163)	1.000
Gender	0.05***	(0.042,0.058)	0.423	0.057***	(0.044,0.07)	0.399
Caste and tribe	0.007***	(0.003,0.011)	0.062	0.015***	(0.007,0.023)	0.102
Wealth	0.008***	(0.004,0.012)	0.069	0.019***	(0.008,0.029)	0.130
Education	0.016***	(0.01,0.022)	0.135	0.012***	(0.004,0.019)	0.083
Location of living	0.037***	(0.029,0.045)	0.312	0.041***	(0.027,0.054)	0.284
**Age 30-39**						
Dissimilarity index	0.09***	(0.084,0.097)	1.000	0.144***	(0.131,0.157)	1.000
Gender	0.023***	(0.018,0.028)	0.255	0.053***	(0.044,0.061)	0.365
Caste and tribe	0.009***	(0.006,0.012)	0.103	0.02***	(0.013,0.027)	0.140
Wealth	0.021***	(0.017,0.025)	0.231	0.022***	(0.014,0.029)	0.150
Education	0.008***	(0.006,0.01)	0.087	0.009***	(0.003,0.015)	0.063
Location of living	0.029***	(0.024,0.034)	0.325	0.041***	(0.03,0.051)	0.282
**Age 40-49**						
Dissimilarity index	0.08***	(0.074,0.085)	1.000	0.175***	(0.164,0.186)	1.000
Gender	0.005***	(0.003,0.007)	0.067	0.027***	(0.019,0.034)	0.152
Caste and tribe	0.01***	(0.008,0.013)	0.127	0.023***	(0.018,0.028)	0.134
Wealth	0.027***	(0.024,0.031)	0.345	0.063***	(0.055,0.072)	0.363
Education	0.012***	(0.01,0.015)	0.155	0.023***	(0.018,0.028)	0.132
Location of living	0.024***	(0.021,0.028)	0.306	0.038***	(0.032,0.045)	0.220

Note: 95 percent confidence intervals (CIs) are calculated by bootstrap with 200 repetitions. Contributory categories are defined as follows: 1) Gender: male (binary variable), 2) Caste and Tribe: scheduled caste, scheduled tribe, and other backward class, 3) Wealth: wealth quintiles, 4) Education: primary, secondary, and higher educational achievement, 5) Location of living: urban and regional (binary variables). Pre-pandemic period: January 20, 2015 - December 04, 2016. $^{*}p < 0.1, ^{**}p < 0.05, ^{***}p < 0.01$
