# Supplementary material for: Socio-economic gradients in hypertension and diabetes management amid the COVID-19 pandemic in India
Source: PLoS One. 2025 Apr 2;20(4):e0315867. doi: 10.1371/journal.pone.0315867 (PMC11964223; doi:10.1371/journal.pone.0315867)
Supplement: Attachment — Submitted filename: Reply to reviewers.pdf [file pone.0315867.s002.pdf]

Reply to reviewers' comments

> Reviewer #1: The article is well-written and addresses a critical issue about access to medication for diabetes and hypertension before and during the pandemic. The study aims to understand the effect of the pandemic on the socioeconomic inequalities in access to medication. Although the study carries out an extensive empirical analysis, it does not reveal the effects of the pandemic. Also, the study needs to include a comprehensive literature survey. Detailed comments about both points are given below:

(Reply)

Thank you very much for reviewing my paper and offering valuable insights and suggestions. Your feedback has greatly enhanced the quality of the manuscript. I have highlighted the key revisions in the updated manuscript, and my detailed responses to your comments are outlined below.

>1. The study uses NFHS-5 dataset for India. This survey was conducted in two phases due to the pandemic-related disturbance in the initial survey schedule. As a result, the two phases cover different districts/states. So, the observed differences in the socioeconomic inequalities in access to medication may result from inherent differences in these districts and not due to the pandemic. The author can utilize NFHS-4 data to make the comparison using the difference-in-difference method.

(Reply)

Thank you for your comment and suggestion. Since the western regions were not surveyed during the pandemic period, they are also excluded from the analysis for the pre-pandemic period to ensure full comparability. This exclusion mitigates potential bias stemming from non-comparability due to unobservable regional differences.

As you suggested, NFHS-4 can be used to provide evidence for the pre-pandemic period. One advantage is that it allows for a larger sample size when estimating the D-index before the pandemic. However, in NFHS-4, only men aged 15–49 and women aged 15–54 were surveyed, and the survey did not inquire about diabetes medication.

Therefore, the revised manuscript provides additional results only on hypertension and its medication in NFHS-4 solely for reference purposes in the Appendix.

>2. A comprehensive review of existing studies is required as some crucial references are missing. For example,

Press, J. · Manne-Goehler, J. · Jaacks, L.M. · et al. Hypertension screening, awareness, treatment, and control in India: a nationally representative cross-sectional study among individuals aged 15 to 49 years PLoS Med. ; 16 (2019), e1002801

Rao Guthi, Visweswara et al. 2024. Hypertension treatment cascade among men and women of reproductive age group in India: analysis of National Family Health Survey-5 (2019–2021) The Lancet Regional Health - Southeast Asia, Volume 23, 100271 (2024).

Maiti, S., Akhtar, S., Upadhyay, A.K. et al. Socioeconomic inequality in awareness, treatment and control of diabetes among adults in India: Evidence from National Family Health Survey of India (NFHS), 2019–2021. Sci Rep 13, 2971 (2023). https://doi.org/10.1038/s41598-023-29978-y

(Reply)

Thank you for letting me know the important literature. In the revised manuscript, they are included in the literature review. I've also substantially updated the literature review by adding a few more studies about the socioeconomic inequality in hypertension and diabetes treatment.

>3. Lastly, the authors should bring out the contribution of this study in light of the literature.

(Reply)

Thank you for your comments. In the revised draft, I've extended the discussion and

explicitly clarifies the contributions of this research to the existing literature at the end of the Introduction section. Especially, I discussed why analysing inequalities in healthcare use during the pandemic is important. Furthermore, the extended implications added in the discussion section also highlights the contribution of this research.

>Reviewer #2: This paper examines the socioeconomic disparities in hypertension and diabetes prevalence and treatment in India before and during the COVID-19 pandemic. Using two waves of a nationally representative populations survey, the authors employ a Shapley decomposition analysis to identify the drivers of disadvantage and undertreatment of these illnesses. It is a relevant to distill the relative impact of demographic characteristics on health status and the analysis is technically sound. However, in its current form, the analysis makes a limited contribution to what is already know about this topic.

(Reply)

Thank you very much for reviewing my paper and offering valuable insights and suggestions. Your feedback has greatly enhanced the quality of our work. I have highlighted the key revisions in the updated manuscript, and my detailed responses to your comments are outlined below.

> 1. This paper could benefit by situating the issues in the context of the Indian health system and health policies. More details about NCD policies, different care providers, and levels of care seeking could help motivate and specify the research question. Specifically, are outcomes ameliorating for some demographic subgroups compared to others? These details would help motivate the current research question. But in its current form, the novelty of the findings seem limited.

(Reply)

Thank you for your valuable comments. In the revised draft, I have restructured the introduction and added a new section that provides information on the healthcare system in India and its challenges. I have also discussed why socioeconomic inequalities in healthcare access occur and why addressing them is essential. Additionally, I have updated the literature review and clarified the contribution of this study. I hope these revisions effectively motivate the current research question.

>2. Relatedly, the discussion section could go much further in discussing the policy and reform implications of the findings. Specifically, what has been tried so far to address the growing burden of hypertension and diabetes, and how do these findings address their limited success thus far?

(Reply)

In the revised manuscript, I have discussed the ongoing comprehensive policy called the National Programme for Prevention and Control of Cancer, Diabetes, Cardiovascular Diseases, and Stroke (NPCDCS), launched by the Indian government in 2010. While this policy aims to address the growing burden of non-communicable diseases (NCDs), recent studies highlight its limited success due to a shortage of both physical and human resources. I have emphasized the importance of overcoming these supply-side challenges to mitigate the worsening socioeconomic inequality in access to treatment. I hope the newly added paragraphs in the discussion section provide necessary direction toward ensuring universally accessible treatment for all, regardless of socioeconomic background.

>3. As the authors mention, there is an extensive literature documenting the demographic determinants of NCDs globally. Yet in its current form, the manuscript has little purchase to answer new questions about the persistence and growing prevalence of NCDs in India.

(Reply)

Thank you for your comment. In the revised draft, I have discussed the distinct roles of public and private healthcare facilities in India and why socioeconomic inequality in healthcare persists. By focusing on the exogenous COVID-19 shock, this study provides new evidence on the exacerbation of socioeconomic inequality. To clarify the contribution of this study, I have added a new subsection outlining the trend of healthcare use for NCDs during the pandemic period.

Additionally, I have explicitly highlighted the research contribution to the literature and emphasized the value of analysing the sources of socioeconomic inequality in

healthcare access, particularly in the context of the COVID-19 pandemic.
