# Supplementary material for: Socio-economic gradients in hypertension and diabetes management amid the COVID-19 pandemic in India
Source: PLoS One. 2025 Apr 2;20(4):e0315867. doi: 10.1371/journal.pone.0315867 (PMC11964223; doi:10.1371/journal.pone.0315867)
Supplement: Attachment — Submitted filename: Response letter.pdf [file pone.0315867.s003.pdf]

Response to editor’s comments:

>As reviewer 1 pointed out, NFHS 5 was carried out in different districts/states in the 2 phases and any observed differences in the outcomes may not be attributable to the pandemic. Consequently, one must be careful about the conclusions on ‘how socioeconomic gradients shifted during the pandemic’ based on such comparisons [aim 2 of the study].

(Reply)

Thank you for your valuable feedback. As you mentioned, the observed difference may not have been caused directly by the pandemic itself. In the revised manuscript, I have clarified that this difference does not necessarily reflect the causal effect of the pandemic but rather shows the association between socioeconomic inequality in treatment and the pandemic period.

I have also revised the description of the study's second aim to avoid any potential impression that this study estimates the causal impact of the pandemic on inequality.

> Second, the study explores the socioeconomic gradients and exploring the underlying drivers of the observed inequality during the pandemic. Excluding the western regions does not solve the problem because of 2 reasons. First, there are many other states that were not surveyed during the pandemic period but have not been dropped from your analysis. Second, given health falls under the jurisdiction of the India states (not the federal government), inter-state/district comparisons (using difference-in-difference methods or state/district fixed effects) are common in the literature. Even if you retain only those states that were surveyed in both phases, do inter-state comparisons in the Indian context make sense? For example, can we compare the outcomes in Haryana to those in Tamil Nadu? This must be explained or clearly discussed in the manuscript.

(Reply)

Thank you for your comments. In the previous draft, I excluded the western region, as it was the only region with states completely missing during the pandemic period. However, as you noted, this may not have been sufficient. In the revised draft, I have included only those states that were surveyed in both the pre-pandemic and pandemic periods to ensure comparability. The limited generalizability of the result due to this sample selection is acknowledged in the discussion. I re-ran the analysis and reinterpreted the results, which now show a worsening of socioeconomic inequalities in the treatment of hypertension and diabetes among middle-aged individuals.

To address potential differences across states, my analysis controls for state fixed effects, and I have clarified this in the revised draft. Since the pandemic affected the entire country, a difference-in-differences approach would not be appropriate in this context, as there are no control states unaffected by the pandemic. Your suggestion is appreciated.

Given that this analysis focuses on a nationwide, aggregated measure of socioeconomic inequality, inter-state comparisons are not the objective. The dissimilarity index calculated in this study reflects inequality in the probability of receiving treatment at the national level. Although it would technically be possible to estimate the dissimilarity index for each state, this study avoids this due to limited sample sizes in some states, which would impede accurate calculation and exploration of determinants. I have discussed this issue in the revised manuscript.

>There is also a need for greater clarity on the contributions of the study relative to the existing literature, especially based on how you decide to address the above concern. It would be great to understand what the value add of this study is over the Rao Guthi, Visweswara et al. 2024 and Maiti et. Al, 2023 papers. Is the difference between the studies in terms of the samples (all states in the cited paper vs. during pandemic in the current study)? Or does the current study have methodological advantages over the existing ones?

(Reply)

Thank you for your suggestion. The strength of my study lies in the direct quantification of horizontal socioeconomic inequality in NCD treatment using the dissimilarity index and its decomposition analysis. This approach offers an advantage over traditional regression analysis by accounting for the entire distribution of treatment accessibility.

In the revised manuscript, I have included the following discussion to highlight the added value of this approach to the existing literature:

> *"First, it quantifies socioeconomic inequalities in the management of hypertension and diabetes using the dissimilarity index (D-index). Unlike a dichotomous regression approach, which estimates socioeconomic inequality based on the relationship between a binary treatment status and its determinants, the D-index reflects the overall distribution of the predicted probability of receiving treatment across the population. For example, if we are interested in disparities in hypertension treatment between educated and less-educated groups, traditional regression analysis would estimate the average difference in treatment rates between these two groups. While regression analysis might indicate that, on average, less-educated individuals have higher access to treatment than more-educated individuals, it does not reveal where within the distribution these disparities are most pronounced. In other words, regression provides a broad indication of inequality but does not show how treatment is distributed within each group. The dissimilarity index, on the other hand, captures absolute differences in treatment across the entire distribution by measuring the share of individuals who would need to switch treatment status to achieve equality between groups. Visual analysis of the D-index can further reveal whether disparities are pervasive across all levels or concentrated at specific levels, offering a more comprehensive view of the structure of socioeconomic inequality."*

I hope this revision clarifies the unique contributions of this approach.
