# Supplementary material for: Socio-economic gradients in hypertension and diabetes management amid the COVID-19 pandemic in India
Source: PLoS One. 2025 Apr 2;20(4):e0315867. doi: 10.1371/journal.pone.0315867 (PMC11964223; doi:10.1371/journal.pone.0315867)
Supplement: Attachment — Submitted filename: Response letter.pdf [file pone.0315867.s005.pdf]

Response to editor’s comments:

Thank you for your valuable suggestions. I have addressed all the comments provided in my draft. Thanks to your feedback, the revised draft has been significantly improved. My responses are summarized as follows.

>Please re-phrase or replace the hyphen with a comma; easy to mistake the hyphen as indicating a range.

(Reply)
I have changed it.

>The references you cite are mostly disease or country specific. Please consider the following references (and others if necessary) to better substantiate your claim:

Roy, Charlotte M., et al. "Assessing the indirect effects of COVID-19 on healthcare delivery, utilization and health outcomes: a scoping review." European Journal of Public Health 31.3 (2021): 634-640.

Moynihan, Ray, et al. "Impact of COVID-19 pandemic on utilisation of healthcare services: a systematic review." BMJ open 11.3 (2021): e045343.
(Reply)
Thank you for your advice. I have reorganized the references and incorporated the suggested literature.

>There are now several peer-reviewed studies to substantiate your claim. Please include a few of those.
(Reply)
I have incorporated relevant studies to strengthen my argument.

>Please include references for the supply side effects of the restrictions
(Reply)
I have incorporated relevant studies to strengthen my argument.

>Please check for accuracy. Sub-district hospitals also provide secondary care along with DHs.
(Reply)
Thank you for pointing that out. I have revised the sentence as follows:
"At the secondary level, district hospitals (DHs), equipped with approximately 200 beds, cater to populations of 1--2 million and complement the CHCs in providing secondary care."

>You reference a study from 2009. Please use an updated reference
(Reply)
Thank you for your suggestion. I have cited a more recent article.

>Please clarify who are the unit of analysis. Is it all adults over 18 yr for the hypertension & diabetes analysis but men and women of eligible ages for the medication analysis?

(Reply)
For clarity, I have added further explanation as follows:
"Accordingly, all adults aged 18 years and older constitute the sample analysed in this study. For the medication analysis, only those aged 18 years and older who are identified as hypertensive or diabetic are included."

>Please summarize the findings from Fig 2 and 3. If these do not provide any new information, please consider moving to the appendix.
(Reply)
I have moved them to the appendix as suggested.

>It will be helpful for readers if you briefly summarize the results from the pre-pandemic period.
(Reply)
Thank you for your suggestion. I've added a few sentences to summarize the results.

> Correct the title of Table 5
(Reply)
Thank you for pointing this out. I've corrected the title.

> Please consider adding a few lines on the comparison of results before and during the pandemic. This needs to be dealt with consistently throughout the paper since you are presenting results from both periods.

(Reply)

Thank you for your suggestion. I have included the following argument:

"Moreover, a comparison of the regression and decomposition results before and during the pandemic indicates that the worsening socioeconomic inequality in medication was closely associated with an increased wealth gradient. This underscores the greater importance of providing social support to less wealthy households requiring treatment to achieve horizontal equity in healthcare access during emergencies."
